# Systematic Analysis of tRNA-Derived Small RNAs Discloses New Therapeutic Targets of Caloric Restriction in Myocardial Ischemic Rats

**DOI:** 10.3389/fcell.2020.568116

**Published:** 2020-11-03

**Authors:** Wenjing Liu, Yang Liu, Zhaohai Pan, Xin Zhang, Yao Qin, Xiaojie Chen, Minjing Li, Xiaoyu Chen, Qiusheng Zheng, Xiaona Liu, Defang Li

**Affiliations:** ^1^Yantai Key Laboratory of Pharmacology of Traditional Chinese Medicine in Tumor Metabolism, School of Integrated Traditional Chinese and Western Medicine, Binzhou Medical University, Yantai, China; ^2^Department of Cardiology, Fuwai Hospital, National Center for Cardiovascular Diseases, Chinese Academy of Medical Sciences, Peking Union Medical College, Beijing, China; ^3^Key Laboratory of Xinjiang Endemic Phytomedicine Resources, Ministry of Education, School of Pharmacy, Shihezi University, Shihezi, China

**Keywords:** caloric restriction, tsRNA, myocardial ischemia, sequencing, bioinformatics analysis

## Abstract

Caloric restriction (CR) is a novel dietary therapy that has a protective effect on myocardial ischemia. However, the mechanisms underlying the therapeutic effect of CR remain unclear. Transfer RNA–derived small RNAs (tsRNAs) are a novel type of short non-coding RNAs that have potential regulatory functions in various physiological and pathological processes. In this study, we explored new therapeutic targets of CR through tsRNA sequencing. Rats were randomly divided into three groups: a normal control group (norm group), isoproterenol (ISO)–induced myocardial ischemic group (MI group), and CR pretreatment plus ISO-induced myocardial ischemic group (CR + MI group). Triphenyl tetrazolium chloride staining, terminal deoxynucleotidyl transferase dUTP nick-end labeling staining, serum creatine kinase (CK) and lactic acid dehydrogenase activity detection kits, and creatine kinase isoenzyme 1 levels were used to measure the degree of myocardial ischemic injury. These indicators of myocardial ischemia were significantly improved in the CR + MI group compared with those in the MI group. In the ischemic myocardial tissue of the MI group, a total of 708 precisely matched tsRNAs were identified, and 302 tsRNAs (fold change >1.5, *P* < 0.05) were significantly changed when compared with those in the norm group. Furthermore, 55 tsRNAs were significantly regulated by CR pretreatment, among which five tsRNAs (tiRNA-His-GTG-004, tRF-Gly-TCC-018, tRF-Cys-GCA-022, tRF-Lys-CTT-026, tRF-Met-CAT-008) were randomly selected and verified by quantitative real-time polymerase chain reaction. In addition, predictions of target genes and bioinformatics analysis indicated that these tsRNAs may play a therapeutic role through the regulation of macromolecular metabolism. In conclusion, our findings reveal that tsRNAs are potential therapeutic targets for CR pre-pretreatment to improve myocardial ischemic injury. This study provides new ideas for future research on elucidating the mechanisms of CR pretreatment in ameliorating myocardial ischemic injury.

## Introduction

Ischemic heart disease is a widespread public health problem, and cardiovascular disease is the leading cause of death worldwide (Wong et al., 2017). Myocardial ischemia refers to a decrease in blood perfusion of the heart, resulting in a decrease in oxygen supply to the heart, abnormal myocardial energy metabolism, and a pathological state that cannot support the normal functioning of the heart (Li et al., 2012). When myocardial ischemia occurs, it also induces hypoxia by decreasing the ability to scavenge oxygen free radicals, as well as increases lipid peroxidation that causes severe damage to vascular endothelial cells (Khan et al., 2018). At this time, myocardial zymograms exhibit abnormal signatures, and levels of serum lactic acid dehydrogenase (LDH) and creatine kinase (CK) are significantly reduced (Li et al., 2012). The main features of myocardial ischemia include myocardial hypoxia, imbalance of energy metabolism (Niemann et al., 2018), cardiac dysfunction, and cardiomyocytic apoptosis (Sung et al., 2017). Clinically, myocardial ischemia can manifest as angina pectoris, myocardial infarction, arrhythmia, and even sudden death. Moreover, myocardial ischemia is one of the most common diseases that endanger public health (Rezende et al., 2019). Hence, considerable research has been dedicated to elucidating its pathogenesis and in identifying and developing preventive treatments. Studies have shown that the occurrence and development of myocardial ischemia and related injuries are closely related to vascular endothelial dysfunction and inflammatory responses (Muliterno et al., 2014), mitochondrial dysfunction (Xue et al., 2017), oxygen free radical damage (Chen et al., 2016), and cardiomyocytic apoptosis (Kobayashi et al., 2018). In order to further ameliorate myocardial damage caused by myocardial ischemic injury, novel treatment strategies are urgently needed.

Caloric restriction (CR), a dietary regimen consisting of periodic reductions in food intake, has been demonstrated to promote protection against diabetes, cancer, heart disease, and neurodegeneration (Brandhorst et al., 2015). Furthermore, CR has also been shown to induce a rapid protective effect against ischemic renal and liver injury (Mitchell et al., 2010). Interestingly, CR has been reported to regulate neural network activity by optimizing peripheral energy metabolism, which enhances parasympathetic activities of autonomic neurons innervating the heart and arteries, thereby reducing heart rate and blood pressure while concomitantly increasing stress resistance and heart rate variability (Longo and Mattson, 2014). Additionally, the myocardial postischemic inflammatory response has been shown to be attenuated by CR reducing oxidative stress in rat models of myocardial ischemia (Ahmet et al., 2005), during which the heart is protected against ischemic injury (Chandrasekar et al., 2001). Nevertheless, a lack of appropriate methods has resulted in the complex regulatory mechanisms of CR ameliorating myocardial ischemia still not been fully understood.

Non-coding RNAs (ncRNAs) play important roles in gene regulation and are involved in many different disease pathogeneses (Hu et al., 2019). Specifically, a large number of transfer RNA (tRNA)–derived small RNAs (tsRNAs, also known as tRNA-derived RNA fragments [tRFs]) have been unveiled with the evolution of high-throughput sequencing technology (Kumar et al., 2016). These tsRNAs, as a class of ncRNAs (18–40 nt in length), are crucial for protein synthesis both in prokaryotic and eukaryotic cells (Jacquier, 2009; Zhu et al., 2018). Many studies have indicated that tsRNAs are not random degradation products of tRNAs but instead perform important roles in many biological activities, as tRFs exhibit similar functions to those of microRNAs (miRNAs) and regulate mRNA stability (Maute et al., 2013). A recently study demonstrated that ncRNAs represent important biomarkers during the processes of cardiovascular diseases because of their stabilities in blood and bodily fluids (Mathiyalagan et al., 2014). Consequently, in view of the cardioprotection of CR during myocardial ischemic processes (Ahmet et al., 2005), we hypothesized that CR may confer therapeutic actions via tsRNAs.

**FIGURE 1:**
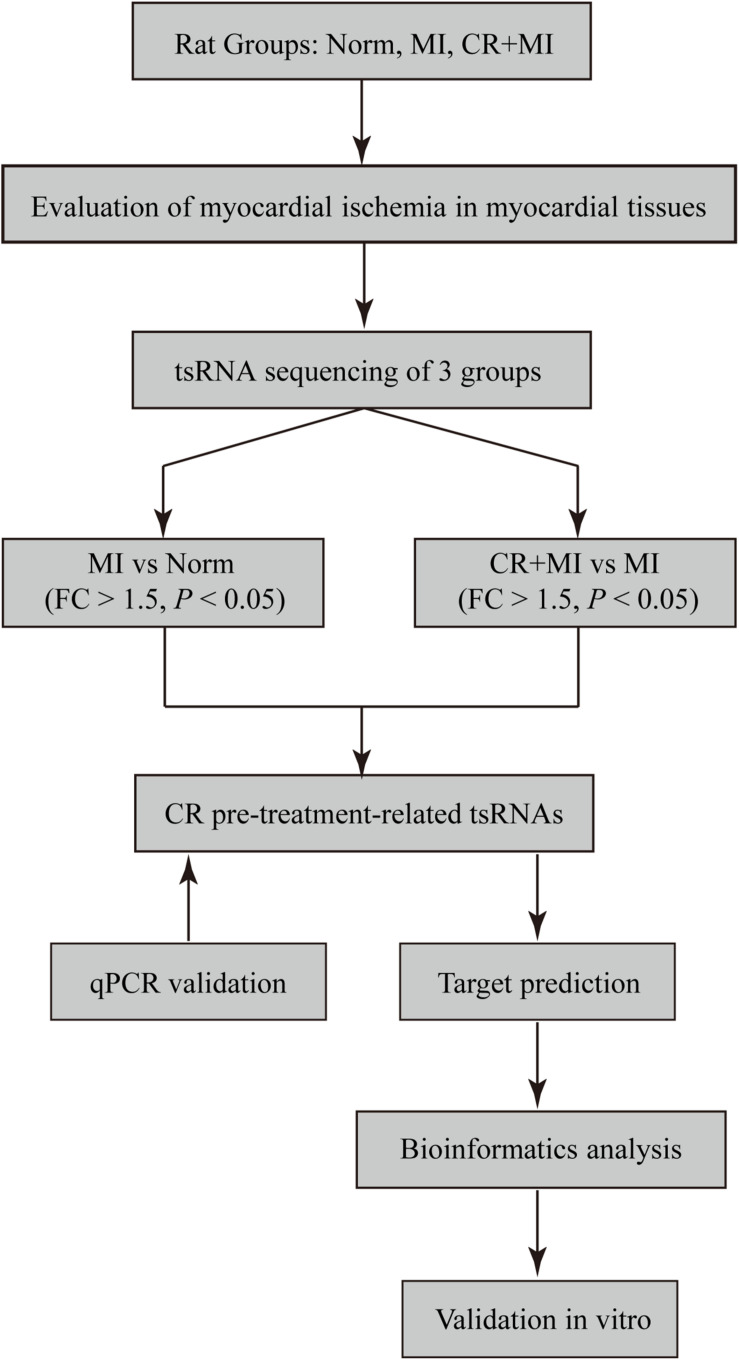
Study design. Norm, normal control group; MI, isoproterenol-induced myocardial ischemic group; CR, caloric restriction; CR + MI, CR pretreatment plus ISO-induced myocardial ischemic group.

In the present study, we used high-throughput RNA sequencing (RNA-seq) technology to estimate the expression levels of tsRNAs in isoproterenol (ISO)–induced myocardial ischemic rats with/without CR pretreatment. Moreover, the biological functions of five tsRNAs were evaluated to reveal potential therapeutic mechanisms of CR in ameliorating myocardial ischemia (Figure 1). Taken together, our findings may provide a novel perspective for elucidating the molecular mechanisms of CR in providing myocardial protection following myocardial ischemia.

## Materials and Methods

### Experimental Animals and Diets

Adult male Sprague–Dawley rats (8 weeks old; 180 ± 10 g) were originally purchased from Jinan Pengyue Experimental Animal Breeding Co., Ltd. (Jinan, China). All animals were maintained in a pathogen-free environment and were housed in clear accumulated cages, in groups of six animals per cage, at a constant temperature/humidity, 12/12-hour light–dark cycle, and food and water were provided *ad libitum*. At 9 weeks of age, animals were randomly divided into the following three groups: (1) norm group, in which the rats were fed *ad libitum* with a regular diet containing 15.6 kJ/g of energy (3.9 kJ/g protein, 2.6 kJ/g fat, and 9.1 kJ/g carbohydrate); (2) MI group, in which the rats were fed *ad libitum* with a regular diet; and (3) CR + MI group, in which the rats were fed a calorie-restricted diet. The CR + MI group was subjected to its unique dietary regimen for four feeding cycles by feeding rats 50% of the standard daily calorie intake (7.8 kJ/g of energy, containing 0.5 kJ/g protein, 5.0 kJ/g fat, and 2.3 kJ/g carbohydrate) at day 1, approximately 10% of the standard daily calorie intake (1.5 kJ/g of energy, containing 0.01 kJ/g protein, 0.01 kJ/g fat, and 1.48 kJ/g carbohydrate) at days 2–4, and then being provided food *ad libitum* at days 6–10 (Cheng et al., 2017). Experiments and procedures were performed according to the protocols approved by the Ethics Committee for the Care and Use of Animals at Binzhou Medical University.

### Induction of Myocardial Ischemic Injury

After four cycles of CR, myocardial ischemic injury was induced in the rats in the MI and CR + MI groups by subcutaneous injection of 60 mg/kg of ISO hydrochloride (cat. no. I0260, J&K Scientific, Beijing, China), which was prepared by dissolution in physiological saline, for two consecutive days.

### Evaluation of Myocardial Ischemia

The rats from the norm, MI, and CR + MI groups were deeply anesthetized by intraperitoneal injection of sodium pentobarbital (1.0%, 40 mg/kg) on the third day after the injection of ISO, and blood samples were then taken from the inferior vena cava of the rats to measure the activities of serum CK and LDH, and the serum levels of creatine kinase isoenzyme 1 (CK-MB1). The activities of serum CK and LDH were determined by CK assay kit (Nanjing Jiancheng Bioengineering Institute, cat. no. A032-1-1) and LDH activity detection kit (Solarbio, cat. no. BC0685). The serum CK-MB1 levels were measured by rat CK-MB1 ELISA kit (Solarbio, cat. no. SEKR-0059). The heart of each rat was excised immediately and weighed after the end of the blood collection, after which it was washed with physiological saline. Then, each sample was frozen in a refrigerator at −20°C for 10 min and was subsequently cut into 2-mm slices. The tissue sections were placed in 1% triphenyl tetrazolium chloride (TTC) staining solution and incubated at 37°C for 20 min in the dark. Finally, the excess staining solution was washed with phosphate-buffered saline. The color change of the sample was observed by the naked eye to evaluate myocardial ischemic injury in the different groups. The heart tissues from three groups were fixed with 4% formaldehyde for 24 h, washed, cut into 4-μm slices, dehydrated, and finally paraffin-embedded. Subsequently, cardiomyocytic apoptosis was detected in strict accordance with the instructions of the colorimetric terminal deoxynucleotidyl transferase dUTP nick-end labeling (TUNEL) apoptotic assay kit (cat. no. C1098, Beyotime). Cells with brown-stained nuclei were indicative of apoptotic cells, as observed under a microscope (DMI3000B, Leica; Leica Microsystems CMS GmbH).

### RNA Extraction

The rats from the three groups were deeply anesthetized by intraperitoneal injection of sodium pentobarbital. The left ventricles from the hearts of each group were collected for the extraction of total RNA. Total RNA was extracted from the left ventricles of rats in the norm, MI, and CR + MI groups for high-throughput sequencing and quantitative real-time polymerase chain reaction (qRT-PCR). TRIzol (Invitrogen life technologies) was added, and the tissue was homogenized with an electric homogenizer. Chloroform was added and centrifuged at 12,000 × *g* for 15 min to dissolve the RNA in the aqueous phase. RNA was precipitated by the addition of isopropanol, and the resultant RNA pellet was then washed with 75% ethanol and dissolved in RNase-free water. The quality and concentration of RNA were determined using a NanoDrop ND-1000. The total optical densities at a 260/280-nm absorbance ratio of all total RNA samples ranged from 1.8 to 2.0. All RNA solutions were then stored at −70°C.

### tsRNA Sequencing

Total RNAs that were extracted from norm, MI, and CR + MI groups were pretreated with rtStar tRF and tiRNA Pretreatment Kits (Arraystar, United States) to remove RNA modifications that interfere with the construction of small RNA-seq libraries. The cDNA was then synthesized and amplified using Illumina’s proprietary RT primers and amplification primers. Subsequently, 134- to 160-bp PCR-amplified fragments (corresponding to small RNAs that were each 14–40 nt in length) were extracted and purified from an agarose gel. The DNA fragments in well-mixed libraries were denatured with 0.1 M of NaOH to generate single-stranded DNA molecules, after which they were loaded onto a reagent cartridge at a 1.8-pM concentration. The sequencing run was performed on a NextSeq system using a NextSeq 500/550 V2 kit (#FC-404-2005, Illumina) according to the instructions of the manufacturer. Sequencing was carried out by running 50 cycles.

### Data Analysis

Raw sequencing data generated from Illumina NextSeq 500 that passed the Illumina chastity filter were used for further analysis. Trimmed reads (trimmed 5′, 3′-adaptor bases) were aligned (allowing for one mismatch only to the mature tRNA sequences). Reads that did not map were aligned (with bowtie software), allowing for one mismatch only to the precursor tRNA sequences. The remaining reads were aligned, allowing for one mismatch only to miRNA reference sequences via miRDeep2. The exactly matched reads were considered to represent tsRNAs. The abundances of tRFs and tiRNAs were evaluated via their sequencing counts and were normalized as counts per million of total aligned reads.

### Target Prediction of Treatment-Related tsRNAs

Transfer RNA–derived small RNAs contain a number of seed sequences that may match the cross-linking central region of the target mRNAs. A growing number of studies have shown that tsRNAs that have miRNA-like functions can lead to the silencing of mRNAs. In this study, we used two common algorithms to predict tsRNA targets, namely, TargetScan^1^ and miRanda^2^. Additionally, to reduce false-positive results, only genes predicted by both algorithms were considered as targets of tsRNAs. The network illustration was visualized with Cytoscape software (version 3.5.1, the Cytoscape Consortium, San Diego, CA, United States).

### Validation via qRT-PCR

To verify treatment-induced changes in tsRNAs via RNA-seq, all tsRNAs were selected, and their expression levels were detected by qRT-PCR. First, total RNA was reverse transcribed into cDNA using the rtStar First-Strand cDNA Synthesis Kit (3′ and 5′ adaptors; Arraystar) according to the manufacturer’s protocol. Then, qRT-PCR amplification was performed using a Quant-Studio 5 Real-Time PCR System (Applied Biosystems) and 2 × PCR master mix (Arraystar). The amplification conditions were as follows: incubation at 95°C for 10 min and then 40 cycles of 95°C for 10 s, 60°C for 60 s, and 95°C for 15 s. The relative tsRNA expression level was calculated using the 2^–Δ^ ^Δ^ ^*Ct*^ method and was normalized to U6 as a housekeeping gene. Specific primers for each gene are listed in Supplementary Table S1. All reactions were performed in triplicate.

In addition, target mRNAs for the treatment of related tsRNAs were also verified by qRT-PCR. One target per tsRNA was randomly selected. GAPDH was used as an internal control to normalize the data.

### Bioinformatics Analysis

To determine the biological functional associations among target genes, the results were analyzed by Gene Ontology (GO) covering three domains: biological process, cellular component, and molecular function. The GO project provides a controlled vocabulary to describe gene and gene product attributes in any organism^3^. Fisher exact test in Bioconductor’s *topGO* is used to find if there is more overlap between the DE list and the GO annotation list than would be expected by chance. The *p* value produced by *topGO* denotes the significance of GO terms enrichment in the DE genes.

### Cell Culture and Transfections

The rat cardiomyocyte cell line, H9c2, was obtained from The Cell Bank of Type Culture Collection of the Chinese Academy of Sciences China (Shanghai, China). The cells were cultured in a sterile cell culture chamber (HF240, HFALFORCE; Shanghai Lishen Scientific Equipment Co., Ltd.) with 95% air and 5% CO_2_ with saturated humidity at 37°C. Rat cardiomyocyte H9c2 cells were subsequently cultured and tested using Dulbecco modified eagle medium high glucose medium (cat. no. SH30022.01, Hyclone) supplemented with 10% fetal bovine serum and 1% streptomycin mixture (cat. no. P1400, Solarbio). Cells at the logarithmic growth phase were seeded into a six-well plate at a density of 2 × 10^5^ cells/well for transfections. The rno-tRF-Met-008 mimic (AGCAGAGTGGCGCAGCGGAAGCGTGCTGGG), rno-tRF-Lys-026 mimic (GCCCGGCTAGCTCAGTCGGTAGAGCATGG GA), rno-tRF-Cys-022 mimic (GGGGGTATAGCTCAGT GGTAGAGCATTTGACT), rno-tiRNA-His-004 mimic (TGAATCTAACAACAGGAAATCAAATTCCTTATTTACCCA), and the negative control (NC; UCACAACCUCCU AGAAAGAGUAGA) were obtained from Shanghai Integrated Biotech Solutions Company Limited. Lipofectamine 3000 (cat. no. L3000015; Invitrogen) was used to transfect the mimics and NC at a final concentration of 100 nmol, according to the manufacturer’s instructions. All groups were performed in triplicate. After 48 h of transfection, total RNA extraction was performed from the transfected cells. The levels of tsRNA-targeted genes were then measured via qRT-PCR. The specific primers are listed in Supplementary Table S1, and the relevant protocols were the same as those described above.

### Statistical Analysis

Results are presented as the mean ± standard error of the mean (SEM). Statistical analysis was conducted via GraphPad Prism (version 7.00; GraphPad Software, Inc., La Jolla, CA, United States). Differences between two groups were determined using Student’s *t*-tests. One-way analysis of variance (ANOVA) was employed for comparisons among three groups. A *P* < 0.05 was defined as being statistically significant.

## Results

### CR Pretreatment Ameliorates Myocardial Ischemic Injury

**FIGURE 2:**
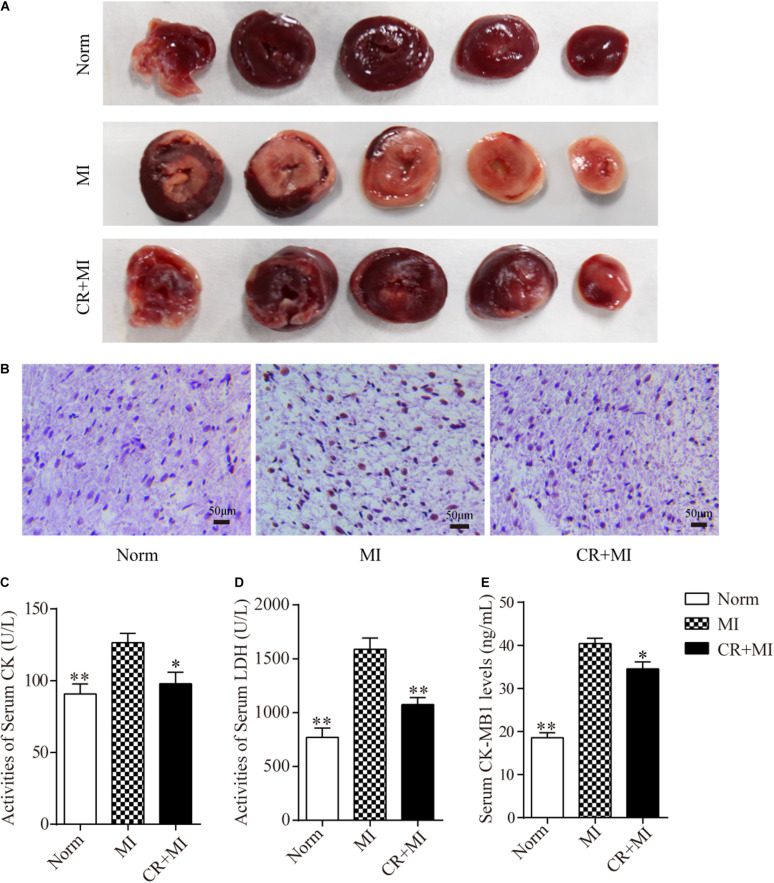
Effects of CR pretreatment on myocardial ischemic injury. **(A)** Representative morphological images of myocardial tissue in norm, MI, and CR + MI groups after TTC staining. **(B)** TUNEL assay of myocardial tissues in norm, MI, and CR + MI groups. Brown staining of the nucleus indicates apoptosis. **(C)** The activities of serum CK were determined by CK assay kit in norm, MI, and CR + MI groups. **(D)** The activities of serum LDH were examined by LDH activity detection kit in norm, MI, and CR + MI groups. **(E)** The levels of serum CK-MB1 were measured by CK-MB1 ELISA kit in norm, MI, and CR + MI groups. The data are presented as the mean ± SEM (*n* = 6). *P < 0.05, **P < 0.01 compared with the MI group.

Triphenyl tetrazolium chloride staining was carried out to detect the effect of CR pretreatment on the area of ischemia in myocardial tissue. The ischemic area in the myocardial tissue in the MI group was significantly larger than that in the norm group, whereas the ischemic area in the myocardial tissue in the CR + MI group was notably reduced compared to that in the MI group (Figure 2A). Furthermore, TUNEL assays revealed that CR pretreatment significantly ameliorated ISO-induced apoptosis compared to that in the MI group (Figure 2B). Next, the activities of serum CK and LDH, and the serum levels of CK-MB1 were used to evaluate whether CR pretreatment reduced ischemic injury after ISO-induced myocardial ischemia. The activities of serum CK and LDH were significantly increased after ISO injection (60 mg/kg) for 2 consecutive days in rats, but the activities of serum CK and LDH were significantly decreased in the CR + MI group compared to those in the MI group (Figures 2C,D). Furthermore, serum CK-MB1 levels were significantly increased in the MI group compared to those in the norm group, and those of the CR + MI group were decreased compared with those of the MI group (Figure 2E).

### Expression Profiles of tsRNA Alterations Induced by CR Pretreatment

**FIGURE 3:**
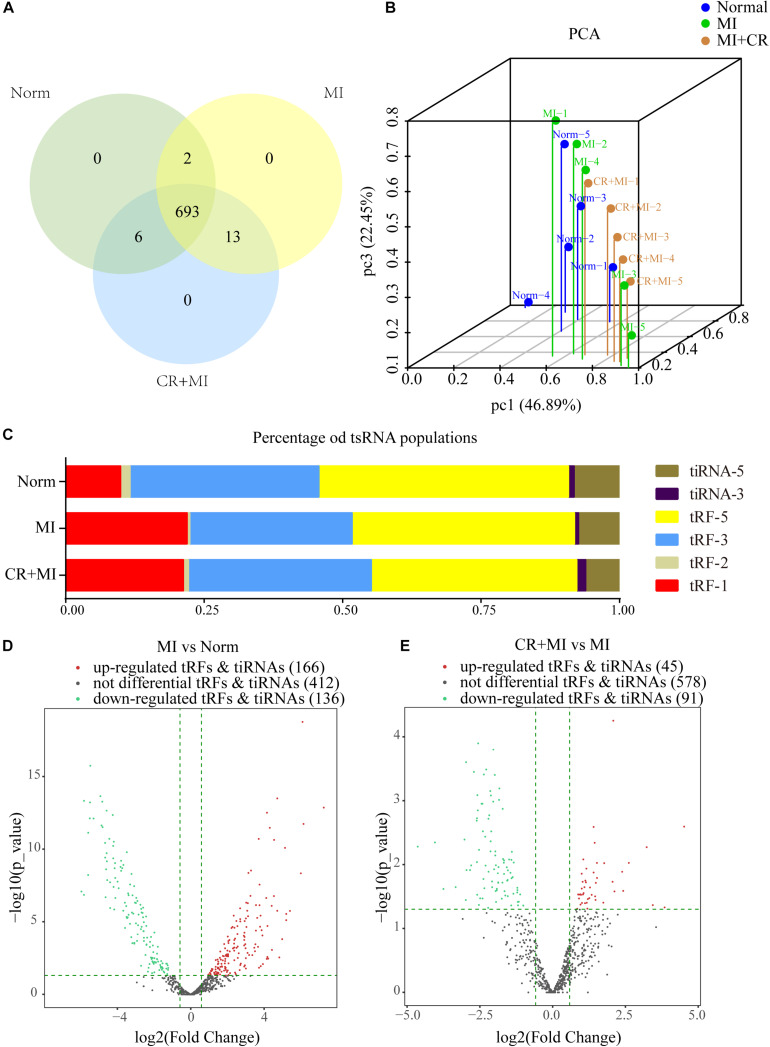
Altered expression profiles of tsRNAs following CR pretreatment. **(A)** Venn diagram indicating the total number of exact-matched tsRNAs in the myocardial tissues of norm, MI, and CR + MI groups. **(B)** PCA plot illustrating the clustering of three repeats in each group and evaluation of the corresponding variability and repeatability (FC > 1.5 and *P* < 0.05). The blue dots represent the norm group, the green dots represent the MI group, and the orange dots represent the CR + MI group. **(C)** Histogram showing the expression levels of each subtype of tsRNA in norm, MI, and CR + MI groups. **(D)** Volcano plot showing 302 significantly altered tsRNAs between the MI and norm groups (FC > 1.5 and *P* < 0.05). **(E)** Volcano plot showing 136 significantly changed tsRNAs between MI and CR + MI groups (FC > 1.5 and *P* < 0.05). Red indicates upregulated tsRNAs, and green denotes downregulated tsRNAs. The data are presented as the mean ± SEM (*n* = 5). *P < 0.05, **P < 0.01 compared with the MI group.

High-throughput sequencing was used to detect differential expression profiles of tsRNAs in the norm, MI, and CR + MI groups (Supplementary Data S1). A total of 714 precisely matched tsRNAs were identified in the myocardial tissues from the three groups of rats (701 in norm, 708 in MI, and 712 in CR + MI) (Figure 3A). In addition, principal component analysis (PCA) was used for unsupervised analysis to reduce the dimensionality of large data sets, which revealed distinguishable tsRNA expression profiles among the 15 samples (Figure 3B).

The expression levels varied among the different tsRNA subtypes, among which more than 90% of the tsRNAs were generated from mature tRNAs, whereas the other 10% derived from pre-tRNAs. Moreover, tRF-3s and tiRNA-3 were decreased in the MI group compared with those in the norm group. Interestingly, CR pretreatment reversed these alterations in the MI group and altered expression profiles such that they were closer to those of the norm group (Figure 3C). In addition, 302 tsRNAs were identified to be notably dysregulated in the MI group: 166 tsRNAs were upregulated, whereas 136 tsRNAs were downregulated compared with those in the norm group. Additionally, after CR pretreatment, we identified 136 tsRNAs that were significantly altered compared with those in the MI group: 45 were upregulated, whereas 91 were downregulated (Figures 3D,E and Supplementary Data S2).

### Targets of CR Pretreatment–Related tsRNAs

**FIGURE 4:**
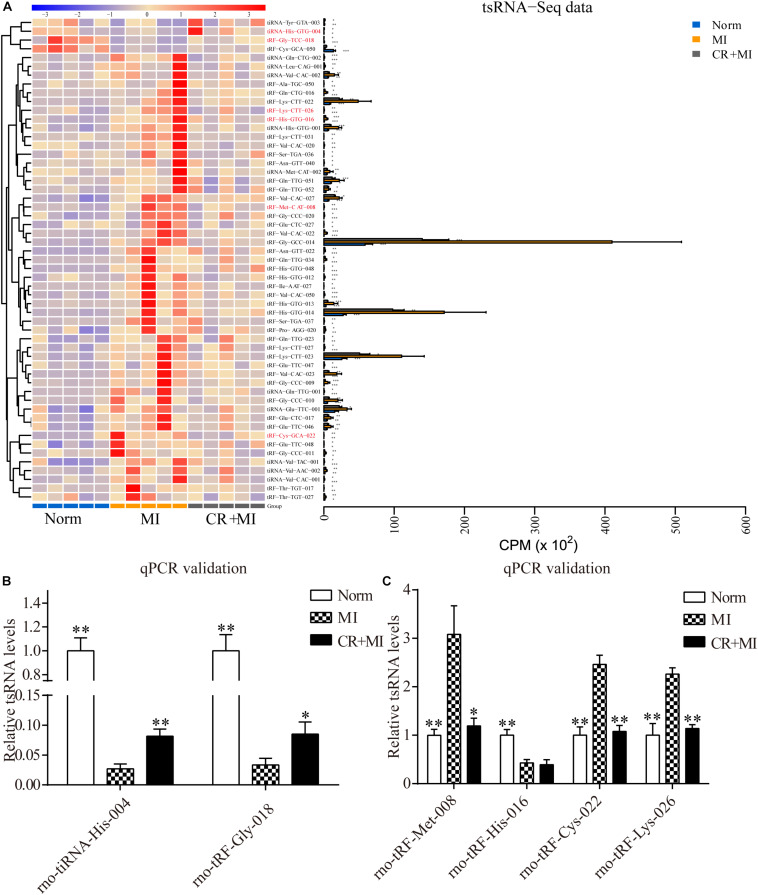
Significantly altered CR-related tsRNAs and their verification via qRT-PCR. **(A)** Significantly altered CR-related tsRNAs are shown in the heat map. The colors in the panel indicate the relative expression levels (log_2_ transformation). The color bar graph on the top panel shows the sample group at the top, and the color bar graph on the right side of the panel represents the partitioning performed using K-means. **(B)** qRT-PCR confirmation of the CR-related tsRNAs, tiRNA-His-GTG-004, and tRF-Gly-TCC-018, among the three groups. **(C)** qRT-PCR confirmation of CR-related tRF-Met-CAT-008, tRF-His-GTG-016, tRF-Cys-GCA-022, and tRF-Lys-CTT-026 among the three groups. The data are presented as the mean ± SEM (*n* = 6). *P < 0.05, **P < 0.01 compared with the MI group.

Hierarchical clustering was performed among the significantly differentially expressed tsRNAs, which indicated distinguishable tsRNA expression profiling among the samples from the three groups. There were 51 tsRNAs that were significantly upregulated in the MI group but downregulated in CR + MI group, whereas four tsRNAs were significantly downregulated in the MI group but upregulated in the CR + MI group (MI vs. norm, CR + MI vs. MI, Figure 4A and Supplementary Data S3). In order to verify the accuracy of the sequencing results, six CR pretreatment–related tsRNAs were randomly identified from all of the altered tsRNAs. Among them, tiRNA-His-GTG-004 and tRF-Gly-TCC-018 were downregulated in the MI group but were upregulated in the CR + MI group. Additionally, tRF-His-GTG-016, tRF-Cys-GCA-022, tRF-Lys-CTT-026, and tRF-Met-CAT-008 were upregulated in the MI group but were downregulated in the CR + MI group (MI vs. norm, CR + MI vs. MI). To verify the results of tsRNA sequencing (tsRNA-seq), qRT-PCR was used to measure changes in the expression levels of the six verified tsRNAs among the three groups. Both tiRNA-His-GTG-004 and tRF-Gly-TCC-018 exhibited significant decreases in the MI group but were increased in the CR + MI group (MI vs. norm, CR + MI vs. MI, Figure 4B). Furthermore, the qRT-PCR results of tRF-Cys-GCA-022, tRF-Lys-CTT-026, and tRF-Met-CAT-008 were consistent with the tsRNA-seq data in that these levels were upregulated in the MI group and downregulated in the CR + MI group (Figure 4C). In contrast, tRF-His-GTG-016 levels were significantly decreased in both the MI and CR + MI groups (MI vs. norm, CR + MI vs. MI, Figure 4C). Based on these results, these five verified CR pretreatment–related tsRNAs were chosen for further analysis.

### Validation of Target Genes of CR Pretreatment–Related tsRNAs

**FIGURE 5:**
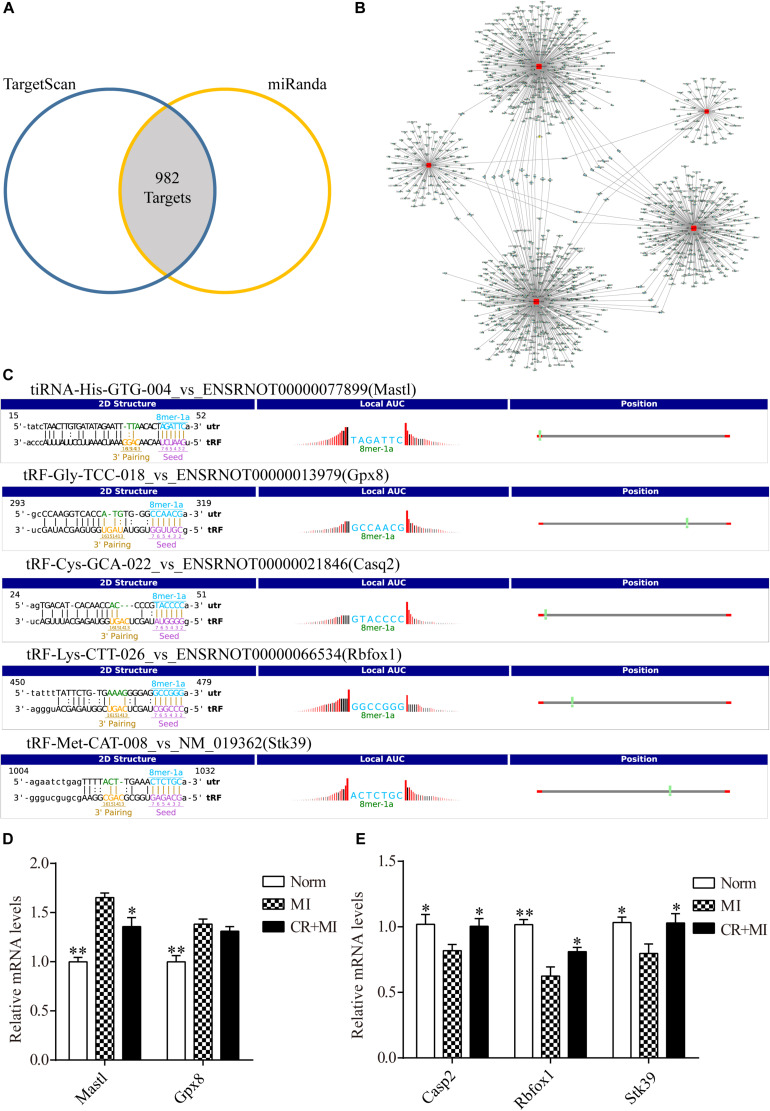
Target genes of CR-related tsRNAs and their verification. **(A)** Venn diagram showing 982 mRNA targets of five CR pretreatment–related tsRNAs that were predicted by two prediction algorithms. **(B)** The target of each tsRNA is shown separately. **(C)** The binding region and seed sequence of five randomly selected mRNA transcripts (each corresponding to a tsRNA sequence). **(D)** Verification of target predictions of Mastl and Gpx8 via qRT-PCR. **(E)** Verification of target predictions of Casp2, Rbfox1, and Stk39 via qRT-PCR. The data are presented as the mean ± SEM (*n* = 6). *P < 0.05, **P < 0.01 compared with the MI group.

Transfer RNA–derived small RNAs recognize target mRNAs and repress their translation via conserved complementary sequence matching. Based on this mechanism, we used two algorithms, TargetScan and Miranda, to predict target genes of tsRNAs. Overall, for five tsRNAs associated with CR pretreatment, 982 mRNA targets were predicted simultaneously (Figure 5A). tiRNA-His-GTG-004, tRF-Gly-TCC-018, tRF-Cys-GCA-022, tRF-Lys-CTT-026, and tRF-Met-CAT-008 were predicted to target 75, 118, 362, 288, and 407 transcripts, respectively (Figure 5B and Supplementary Data S4). In the present study, CR pretreatment–related tsRNAs were reduced in the MI group and increased in the CR + MI group. Therefore, we predicted that their target-gene mRNAs should be increased in the MI group but decreased in the CR + MI group. Otherwise, if CR pretreatment–related tsRNAs were increased in the MI group and reduced in the CR + MI group, their target-gene mRNAs should be downregulated in the MI group and upregulated in the CR + MI group.

First, the expression levels of mRNAs in rat myocardial tissue were detected by qRT-PCR to verify the relationship of expression levels between each tsRNA and its corresponding target gene. The tsRNAs that targeted the following mRNA genes were randomly selected: Mastl, Gpx8, Casp2, Rbfox1, and Stk39. The binding region and seed sequence for each of these tsRNAs are shown in Figure 5C. Consistent with the theoretical results, except for the target gene of tRF-Gly-TCC-018, when the expression levels of tiRNA-His-GTG-004 were downregulated in the MI group and upregulated in the CR + MI group, the expression levels of the corresponding mRNA Mastl were upregulated in the MI group and downregulated in the CR + MI group (Figure 5D). Similarly, when the expression levels of the tsRNAs (tRF-Cys-GCA-022, tRF-Lys-CTT-026, and tRF-Met-CAT-008) were upregulated in the MI group and downregulated in the CR + MI group, the expression levels of the corresponding mRNAs (Casp2, Rbfox1, and Stk39) were downregulated in the MI group and upregulated in the CR + MI group (Figure 5E).

**FIGURE 6:**
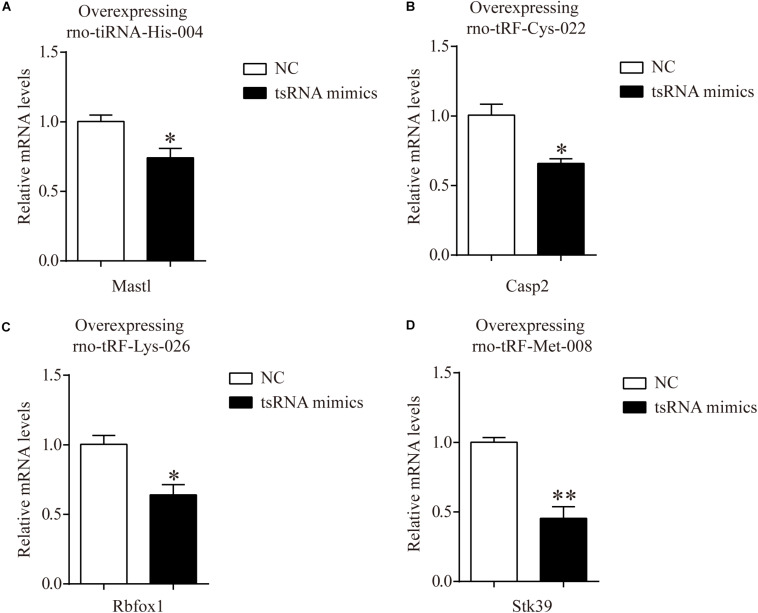
mRNA levels of H9c2 cells transfected with tsRNA mimics. **(A)** The qRT-PCR results of relative mRNA levels in H9c2 cells transfected with rno-tiRNA-His-004 mimics. **(B)** The qRT-PCR results of relative mRNA levels in H9c2 cells transfected with rno-tRF-Cys-022 mimics. **(C)** The qRT-PCR results of relative mRNA levels in H9c2 cells transfected with rno-tRF-Lys-026 mimics. **(D)** The qRT-PCR results of relative mRNA levels in H9c2 cells transfected with rno-tRF-Met-008 mimics. The data are presented as the mean ± SEM (*n* = 3). *P < 0.05, **P < 0.01 compared with NC group.

In addition, to certify the target genes of tsRNAs, we overexpressed four of the candidate tsRNAs (but not tRF-Gly-TCC-018) in H9c2 cells to observe the corresponding changes in tsRNA targets. After transfection with tiRNA-His-GTG-004, the expression of Mastl was significantly downregulated (Figure 6A). The results were the same for Casp2, Rbfox1, and Stk39 when transfected with their corresponding tsRNAs (Figures 6B–D). The results of these *in vitro* experiments are consistent with the results of qRT-PCR in rats, indicating that our target prediction results were reliable and could be used for further analysis.

### tRF Target Genes Transfected in H9c2 Cells Are Enriched in Metabolic Pathways

**FIGURE 7:**
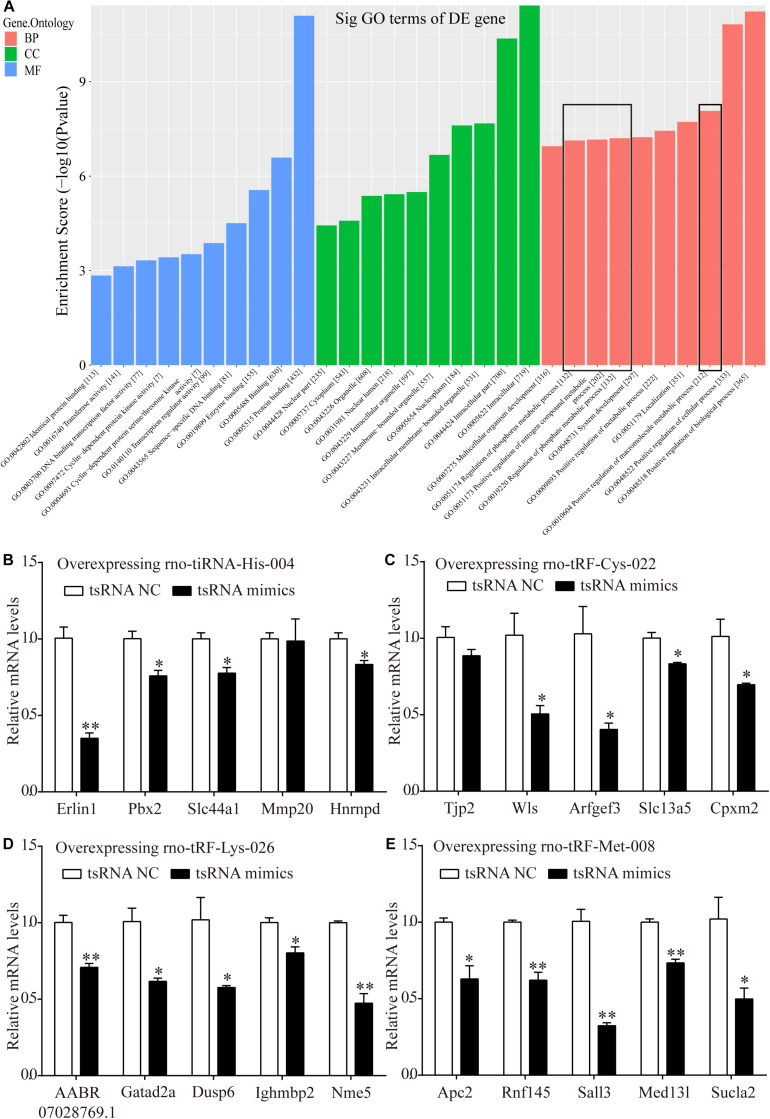
Target mRNAs regulated by four candidate tsRNAs enriched in macromolecular metabolic process and their verification via qRT-PCR. **(A)** The general GO annotations for biological processes, cellular components, and molecular functions of the target mRNAs regulated by the four candidate tsRNAs. **(B)** The relative mRNA levels enriched in macromolecular metabolic process in H9c2 cells transfected with rno-tiRNA-His-004 mimics. **(C)** The relative mRNA levels enriched in macromolecular metabolic process in H9c2 cells transfected with rno-tRF-Cys-022 mimics. **(D)** The relative mRNA levels enriched in macromolecular metabolic process in H9c2 cells transfected with rno-tRF-Lys-026 mimics. **(E)** The relative mRNA levels enriched in macromolecular metabolic process in H9c2 cells transfected with rno-tRF-Met-008 mimics. The data are presented as the mean ± SEM (*n* = 3). *P < 0.05, **P < 0.01 compared with NC group.

Bioinformatics analysis was performed via GO analysis to analyze the functions and putative therapeutic effects of all target mRNAs for each candidate tsRNA. GO cluster analysis was performed for categories including biological processes, cellular components, and molecular functions. The biological processes included the positive regulation of biological processes, cellular processes, macromolecular metabolic processes, metabolic processes, nitrogen-compound metabolic processes, and phosphorus metabolic processes. The classifications of cellular components involved intracellular membrane-bound organelles, nucleoplasms, nuclear lumens, nuclear parts, and cytoplasms. Meanwhile, protein binding, enzyme binding, sequence-specific DNA binding, transcription regulator activity, and DNA-binding transcription factor activity were primarily involved in the molecular functions of the target mRNAs (Figure 7A).

Metabolism is closely related to myocardial ischemic injury. Thus, 20 target genes related to metabolism that were regulated by our four further-analyzed tsRNAs were selected. Erlin1, Slc44a1, Mmp20, Pbx2, and Hnrnpd were regulated by tiRNA-His-GTG-004. Apc2, Rnf145, Sall3, Med13l, and Sucla2 were regulated by tRF-Met-CAT-008. tRF-Cys-GCA-022 regulated Tjp2, Wls, Arfgef3, Slc13a5, and Cpxm2. AABR07028769.1, Gatad2a, Dusp6, Ighmbp2, and Nme5 were targeted by tRF-Lys-CTT-026. Erlin1, Slc44a1, Pbx2, and Hnrnpd were significantly downregulated with the transfection of tiRNA-His-GTG-004 mimics in H9c2 cells, whereas Mmp20 levels were not altered (Figure 7B). In H9c2 cells that were transfected with tRF-Cys-GCA-022 mimics, the expression levels of Wls, Arfgef3, Slc13a5, and Cpxm2 were significantly downregulated, whereas Tjp2 levels were not significantly altered (Figure 7C). The expression levels of the target genes of AABR07028769.1, Gatad2a, Dusp6, Ighmbp2, and Nme5 were significantly downregulated in H9c2 cells after transfection with tRF-Lys-CTT-026 mimics (Figure 7D). The expression levels of Apc2, Rnf145, Sall3, Med13l, and Sucla2 in H9c2 cells were significantly reduced after transfection with tRF-Met-CAT-008 mimics (Figure 7E).

## Discussion

Caloric restriction confers protective effects on ischemic kidney and liver injuries (Mitchell et al., 2010). Furthermore, CR is associated with improved heart remodeling caused by stress overload and yields an increased myocardial ischemic tolerance (Seymour et al., 2006). CR can also reduce inflammatory responses in a rat model of myocardial ischemia and protect the heart from ischemic injury (Chandrasekar et al., 2001; Ahmet et al., 2005). In our present study, TTC staining showed that the myocardial ischemic area of the MI group was increased significantly, while CR pretreatment reversed these changes in the CR + MI group. Therefore, CR pretreatment induced a protective effect on ISO-induced myocardial ischemic injury. TUNEL assays also showed that CR pretreatment ameliorated apoptosis in myocardial tissues, which indicated that CR pretreatment reduced impairment of cardiac function.

In addition, there are many myocardial enzymes (e.g., LDH, CK, and CK-MB) in the myocardium that play an important role in the normal physiological activities of the myocardium (Khalil et al., 2015). These myocardial enzymes are released from myocardial cells and into the serum when pathological conditions such as myocardial ischemia occur, which aggravate the degree of myocardial ischemic damage (Vishwakarma et al., 2018). The results of our present study showed that the activities of serum LDH and CK, and the serum CK-MB1 levels in the MI group were significantly higher than those in the norm group, indicating that subcutaneous injection of ISO caused myocardial ischemia in rats. CR pretreatment significantly reduced the ISO-induced increases in activities of serum LDH and CK and the serum CK-MB1 levels. However, the complex regulatory mechanisms of CR in ameliorating myocardial ischemia have not been fully elucidated. Our present findings reveal that tsRNAs play important roles in CR pretreatment–induced reductions in myocardial ischemia.

Non-coding RNAs play important roles in several physiological and pathological processes of ischemic heart disease and act as potential regulators of myocardial ischemic progression (Guo et al., 2017). ncRNAs have been demonstrated to improve the prognosis of myocardial ischemia by inhibiting cardiomyocytic apoptosis and promoting angiogenesis in animal models, leading to decreases in the ischemic areas in myocardial tissues (Kang et al., 2015; Pei et al., 2015). Our present results of high-throughput sequencing detected differential expression profiles of tsRNAs, which are novel regulatory small ncRNAs that participate in diverse physiological and pathological processes, among our three experimental groups. According to the tsRNA-seq data, five tsRNAs were identified as targets of CR pretreatment, and two of them were significantly downregulated in the MI group but upregulated in the CR + MI group. Meanwhile, the expression levels of three tsRNAs were markedly increased in the MI group but significantly reduced in the CR + MI group (MI vs. norm, CR + MI vs. MI). The verifications of five tsRNAs via qRT-PCR were consistent with our sequencing data. Subsequently, we used bioinformatics to demonstrate the biological functions of the five CR pretreatment–related tsRNAs and further revealed their mechanisms of action.

Transfer RNA–derived small RNAs regulate gene expression. Some studies have shown that tsRNAs inhibit the assembly of the translation initiation machinery to repress translation (Gebetsberger et al., 2017; Guzzi et al., 2018). In contrast, other studies have observed that tsRNAs perform an miRNA-like gene-regulation mechanism associated with argonaute (AGO) proteins (Kumar et al., 2014; Kuscu et al., 2018). tsRNAs have been shown to exhibit tsRNA-mediated transcriptional silencing that is dependent on a 5′ seed, such that the structure of AGO facilitates the 5′ end of small RNAs for target recognition (Boland et al., 2011), which is complementary to target sites within the 3′ UTR (Haussecker et al., 2010). The results of these studies suggest that tsRNAs regulate the expression of genes by targeting various mRNA interactions with other effector proteins or by acting protein-independently (Jehn et al., 2020). Because tsRNAs have only recently been discovered, a database for tsRNA target prediction has not been available. Generally, the target predictions of tsRNA-related mRNAs have no unified algorithm, and the main principle of such predictions is based on miRNA-like methods (Li et al., 2019). Therefore, in the present study, we used two common miRNA algorithms to predict the targets of five CR pretreatment–related tsRNAs, and the roles each tsRNA were inferred by the associated biological functions of the corresponding target mRNA. Considering the reliability of the prediction of tsRNA target genes, we validated such predictions via qRT-PCR. A target mRNA was randomly selected from each tsRNA, and the results of four analyzed tsRNAs were consistent with the predicted results, with the exception of tRF-Gly-TCC-018.

In order to investigate the functions of tsRNA targets, we employed GO analysis. Most of the results were enriched in processes related to myocardial ischemic injury, such as macromolecular metabolism. Energy metabolism disorders and the accumulation of oxygen free radicals and lipid free radicals occurred during the myocardial ischemia (Neri et al., 2015). Previous studies have confirmed that these pathways are involved in the occurrence of myocardial ischemic injury. For instance, a previous study showed that the metabolic responses during myocardial ischemic injury lead to an increase in the mitochondrial citric acid cycle intermediate succinate (Chouchani et al., 2014). Thus, the secretion of succinate may trigger inflammation and neovascularization to contribute to myocardial ischemic injury via mitochondrial ROS (Hamel et al., 2014). Therefore, CR pretreatment may play a therapeutic role in regulating myocardial ischemia–related pathways through tsRNAs.

The pathogenesis of myocardial ischemia is related to energy metabolism (Zhang et al., 2018), arginine metabolism (Rajapakse et al., 2016), and glutamine/glutamate metabolism (Ferdinandy et al., 2014); additionally, macromolecular metabolism is distinctly enriched among these processes and pathways associated with the corresponding target mRNAs. In the present study, we found that the target mRNAs enriched in metabolic signaling pathways were tiRNA-His-GTG-004, tRF-Cys-GCA-022, tRF-Lys-CTT-026, and tRF-Met-CAT-008; their corresponding mRNAs included Med13l, Sucla2, Wls, and so on, all of which are involved in myocardial ischemic injury via influencing metabolism. For instance, recent findings have shown that the Med13l gene is associated with cardiovascular diseases and that the Med subunit modifies glucose and lipid metabolism (Napoli et al., 2019). Furthermore, the increased of Sucla2 induced by Qishen granules may promote the transportation of ATP from mitochondria to cytoplasm, which may contribute to restoring mitochondrial function and facilitating the tricarboxylic acid cycle to exert cardioprotective effects (Gao et al., 2020). Coincidentally, the absence of Wls in myeloid cells in mice yielded improved function and less remodeling after myocardial infarction (Palevski et al., 2017). In our present study, transfection experiments showed that overexpression of four tsRNAs mimics reduced the levels of target genes in metabolic pathways. Therefore, the protective effect of CR pretreatment on myocardial ischemia may be caused by tsRNAs. CR pretreatment induced an increase in tiRNA-His-GTG-004 and reductions in tRF-Cys-GCA-022, tRF-Lys-CTT-026 and tRF-Met-CAT-008, which subsequently affected mRNAs in metabolism-related signaling pathways to confer a therapeutic effect on myocardial ischemic injury. These functions of tsRNAs indicate that tsRNAs may underlie novel and specific therapeutic mechanisms of CR pretreatment on ameliorating myocardial ischemic injury.

Our present study also had some limitations. First, future studies with larger sample sizes and that include clinical samples will be needed to confirm our present results. Second, the specific interactions and the binding sites between tsRNAs and mRNAs require further investigation. Furthermore, the regulatory effect of tsRNAs on the expression levels of ISO-induced differentially expressed myocardial protein molecules (e.g., GRKs or β-arrestins) was not examined in this study.

## Conclusion

Our present study revealed changes in expression profiles of tsRNAs in myocardial ischemic rats after CR pretreatment and implied that tsRNAs could represent therapeutic mechanisms underlying the efficacy of CR pretreatment in ameliorating myocardial ischemic injury. This study may encourage more researchers to further explore the regulatory effect of tsRNAs during myocardial ischemic injury.

## Data Availability Statement

The datasets [the raw data of tsRNAs-Sequencing] for this study can be found in the [NCBI’s Gene Expression Omnibus] [https://www.ncbi.nlm.nih.gov/geo/query/acc.cgi?acc = GSE15 8781].

## Ethics Statement

The animal study was reviewed and approved by the Ethics Committee for the Care and Use of Animals at Binzhou Medical University.

## Author Contributions

WL: data curation, YL: conceptualization, ZP: formal analysis, XZ: methodology, YQ: project administration, XC: investigation, ML: software, XC: resources, DL: validation, XL: visualization, WL, YL, and ZP: writing - original draft, XL, DL, and QZ: writing - review and editing. All authors contributed to the article and approved the submitted version.

## Conflict of Interest

The authors declare that the research was conducted in the absence of any commercial or financial relationships that could be construed as a potential conflict of interest.

## Footnotes

1 http://www.targetscan.org

2 http://www.microrna.org

3 http://www.geneontology.org
