# Supplementary material for: Systematic Analysis of tRNA-Derived Small RNAs Discloses New Therapeutic Targets of Caloric Restriction in Myocardial Ischemic Rats
Source: Front Cell Dev Biol. 2020 Nov 3;8:568116. doi: 10.3389/fcell.2020.568116 (PMC7670042; doi:10.3389/fcell.2020.568116)
Supplement: Supplementary file 1 [file Table_1.docx]

Table S1. Sequences of primers for qPCR validation of candidate genes and internal control.

| Gene name | Primer sequence | Product length |
| --- | --- | --- |
| U6 | F:5’GCTTCGGCAGCACATATACTAAAAT 3’  R:5’CGCTTCACGAATTTGCGTGTCAT 3’ | 89 |
| tiRNA-His-GTG-004 | F:5’ TCCGACGATCTGAATCTAACAAC 3’  R:5’ GCTCTTCCGATCTTGGGTAAAT 3’ | 62 |
| tRF-Gly-TCC-018 | F:5’ GACGATCGCGTTGGTGGTAT 3’  R:5’ CTCTTCCGATCTAGCTATGCTCAC 3’ | 48 |
| tRF-Cys-GCA-022 | F:5’ ACGATCGGGGGTATAGCTCAG 3’  R:5’ CTCTTCCGATCTAGTCAAATGCTCT 3’ | 50 |
| tRF-Lys-CTT-026 | F:5’ TTCTACAGTCCGACGATCGCC 3’  R:5’ CTTCCCATGCTCTACCGACTGA 3’ | 51 |
| tRF-Met-CAT-008 | F:5’ CGATCAGCAGAGTGGCGCA 3’  R:5’ CGATCTCCCAGCACGCTTC 3’ | 41 |
| tRF-His-GTG-016 | F:5’ ATCGCCGTGATCGTATAGTGG 3’  R:5’ GCTCTTCCGATCTACGCAGAGTA 3’ | 49 |
| GAPDH | F:5’ GATGACATCAAGAAGGTGGTGA 3’  R:5’ ACCCTGTTGCTGTAGCCATATTC 3’ | 206 |
| Mastl | F:5’ ACAGTCAGCAAGCAACGTCT 3’  R:5’ GCCATGTCTGTGCAGGTAGT 3’ | 154 |
| Casq2 | F:5’ GGAGCATCAAAGACCCACCC 3’  R:5’ TTCTCCGCAAATGCCACAAT 3’ | 102 |
| Rbfox1 | F:5’ CTACAGTGACAGTTACGGACGAG 3’  R:5’ ATGAAGAAAGAACGAGACCC 3’ | 161 |
| Stk39 | F:5’ CTGGGTAGGAAGGGAGCCT 3’  R:5’ CTTGCCGCCAGGGAAGTTT 3’ | 86 |
| Erlin1 | F:5’ CGTCACTGACCGGAGGAAC 3’  R:5’ GGAGGCGTAAAGGAGGATCG 3’ | 95 |
| Pbx2 | F:5’ CACAAAACAGGGCGATTGGG 3’  R:5’ AAGAAACGGAGACACCCCAC 3’ | 98 |
| Slc44a1 | F:5’ ACACAGCCACAGCCATCAATAGC 3’  R:5’ CAGCCACTCGCAGAGCATTCTC 3’ | 90 |
| Mmp20 | F:5’ CTGGGCCTGGGCCATTCCAC 3’  R:5’ CTGGTGATGGTGCTGGGCCG 3’ | 320 |
| Hnrnpd | F:5’ GCCATTCAAACTCCTCCCCA 3’  R:5’ GCAGTCTACAACGTCCCCAA 3’ | 146 |
| Apc2 | F:5’ ATCCCAAGGCCACCTGGCTA 3’  R:5’ TCCCCACACCGTCACCAAGT 3’ | 223 |
| Rnf145 | F:5’ TGCCATCTGTTACCAGGACAT 3’  R:5’ GACACAGAGGGCAGGTATCC 3’ | 113 |
| Sall3 | F:5’ CTCTTCTTGGTTTCCTAGGCGT 3’  R:5’ TCCGCCCACTTGAAGAACTC 3’ | 130 |
| Med13l | F:5’ TTTACCAAGCAGGTCACGGA 3’  R:5’ ACAATCCCCAGGCACCATTC 3’ | 84 |
| Sucla2 | F:5’ GGACAAAGACGCAGCTGATG 3’  R:5’ AGCCAAGCCAGCACCATTTA 3’ | 85 |
| Tjp2 | F:5’ GCATGTAGACCCAGCAAAGG 3’  R:5’ GGTTTTGTCTCATCATTTCCTCA 3’ | 65 |
| Wls | F:5’ GCTCACTACCACAATCACCCA 3’  R:5’ TCACCTGCCCTGAGGATGAA 3’ | 108 |
| Arfgef3 | F:5’ GCTGTCTAACCCTCTCCAGTC 3’  R:5’ AGATGGGTGTGGGACCTGTA 3’ | 80 |
| Slc13a5 | F:5’ TTCCATCGGCATCCATCCAC 3’  R:5’ GAGGTGTCCGTAGGCAAACA 3’ | 118 |
| Cpxm2 | F:5’ GGCCTATGAAGGAGGTTCCG 3’  R:5’ AACTTTCCTTGGGGCGTTCT 3’ | 142 |
| AABR07028769.1 | F:5’ CCTGTGTACTGACCCACCAC 3’  R:5’ AACAGTGCCCAGAAACCGAA 3’ | 154 |
| Gatad2a | F:5’ TCTACACTTCCTGAGTGGCTGA 3’  R:5’ CCACCCAGTGTTGCTGTTCC 3’ | 83 |
| Dusp6 | F:5’ CAGCAGCGACTGGAATGAGA 3’  R:5’ GCAGTGCAGGGCGAACTC 3’ | 139 |
| Ighmbp2 | F:5’ GTGGCCTTTGATGAGTCCCA 3’  R:5’ GGTCCAGGGTCGTGTTGTAG 3’ | 235 |
| Nme5 | F:5’ TGATTTTGGGACGGGGACTC 3’  R:5’ CTCCATTCTGGCGTTTTCCG 3’ | 85 |
